# Technologies and social services. An overview of technology use by users of social services

**DOI:** 10.1371/journal.pone.0284966

**Published:** 2023-05-04

**Authors:** Rocío Muñoz Moreno, Manuela Fernández Borrero, Elena Ferri Fuentevilla, Fernando Relinque Medina, Aleix Morilla Luchena, Octavio Vázquez Aguado

**Affiliations:** 1 Department of Sociology, Social Work and Public Health, University of Huelva, Huelva, Spain; 2 Department of Social Sciences, Pablo de Olavide University, Seville, Spain; Bucharest University of Economic Studies: Academia de Studii Economice din Bucuresti, ROMANIA

## Abstract

Information and communication technologies have significantly transformed the way advanced societies interact, produce, deliver services and consume resources. All walks of life are now touched by these technologies. However, compared to other areas of society, digital penetration is much lower in the development of and access to social services. The main objective of this paper was to find out what technological devices are used, how they are used and the way citizens interact with public bodies using technology to deliver social services. This has been part of a wider project on innovation in social services using participative methodologies centred on the development of local Hubs. The findings reveal a digital divide in technology-enabled access to social services that excludes the very people most in need of benefits and support.

## Introduction

Digital innovation has been identified as critical to meeting the challenges of today’s society [1]. Digital technology has also been integrated into the professional practice of social work and social services, especially since the global confinement caused by the COVID-19 pandemic. This has brought with it both new developments and new challenges [2]. Since 2020, much has been written and published about “digital social work, describing professional practices using digital media [3, 4], the duty to update digital skills [5, 6], reflections on the ethics, efficiency and viability of such digital practices [7–9] and the opportunities and scenarios envisaged by the technologisation of the profession’s processes [10,11].

In this vein, [1] argues that introducing technology into the field of social services and social intervention can help to streamline workflows, and that “big data” management would allow for the anticipation of needs and early allocation of necessary resources. However, this recognises the potential power imbalances between the various actors and the ethical dilemmas around the protection of privacy and personal data and the violation of the human rights of socially vulnerable people through punitive social control.

The impact of Covid-19, which has been a driver of technological development in social work and social intervention, has brought these challenges to the fore [12].

Turning to those using social services, digital engagement is a complex and multifaceted process involving socio-demographic factors, individual agency [13] and the social context [14]. This study focuses on users of social services, a topic that has been underexplored in the literature and is worthy of special attention given the implications. Some research [13, 14] suggests that people disengage from digital services because of a lack of time for technology; disinterest in digital technology; conservatism; frustration; privacy fears and concerns; lack of access to media, networks and technological resources; and their immediate social environment.

The digital divide is not only generational: it is also socio-economic [15]. In this context, the public sector has an important role to play in stakeholder uptake and use of digital services [16]. Ensuring more democratic access to digital services [17] and preventing the most vulnerable from being excluded [18] requires technologies that are designed to be more accessible and inclusive.

In light of this current, changing, future and challenging landscape, this study aims to help understand the profile of users of local social services in the context of digitalisation and technological integration. We posed the following questions: How do technology-enabled users of social services interact with the system? What is their capacity and willingness to use technology in social services processes? How do they use this technology in their dealings with public bodies? The aim of this paper is to analyse this profile in terms of the availability of resources, as well as users’ knowledge, use and management of new technologies (NTs) in everyday life. Above all, it will look at the use of such technologies in dealing with public bodies and in the administration of various social services and benefits.

## Methodology

### Participants and instrument

This study involved 114 participants who completed the project questionnaire. The sample consists of users of local community social services in different territorial areas of the province of Huelva (Andalusia, Spain). This work complies with the requirements of the Ethics Committee of the Vice-Rector’s Office for Research of the University of Huelva. As it is not experimental research, no specific authorisation document is required. Informed consent was given verbally, informing participants of the purpose of the research. In addition, information on anonymity and the processing of data for statistical purposes was included in the header of the questionnaires. The ethics committee did not require consent.

In terms of the socio-demographic profile, the sample data show that the majority are women (78%), with the most common age groups being 51–65 years old (43%) and 36–59 years old (31.6%), with an average age of just over 50.

With respect to household structure, the majority are couples with children (40.4%) and single-person households (23.7%). The rest is divided between childless couples (13.2%), multi-nuclear households (12.3%) and single-parent households (10.5%). Most households have two members aged 16 or over (34.2%), 23.7% had one member and 20.2% had three members.

In terms of marital status, 39.5% were married, 21.9% were separated or divorced, 21.9% were single, 8.8% were widowed and 7.9% were cohabiting.

With regard to educational attainment, a significant proportion of the population has no education at all (26.3%) or has only a primary education (52.6%). A total of 14.9% have a secondary education and only 6.2% have been educated to higher or postgraduate level. Another important socio-economic factor has to do with employment. A high percentage of the population is unemployed (48.2%), including 22.8% for more than two years. The rest of the sample was either working within the home (19.3%), permanently disabled (8.8%), retired or in early retirement (7.9%) or studying (2.6%). Only 8.8% of the sample was in employment.

More than half (53.5%) of the sample had a household income of 400–800€ and 21% had an income of less than 400€. This means that around 75% of people had a monthly household income of no more than 800€.

It is also important to note that 27.2% of the respondents had a disability. The most commonly reported disability is physical (55%), followed by intellectual, cognitive or developmental disability (34%) and sensory impairment.

An ad-hoc questionnaire was designed to fulfil the study objectives. This was based on various existing instruments, such as the “Survey on Equipment and Use of Information and Communication Technologies in Households” [19] and the work of Ot [20] on social services in the digital era. It also included some questions added by the research team.

With a total of 30 items, most of which are categorical, the final instrument consists of the following dimensions:

- Location data (municipality, neighbourhood, local social services area).
- Socio-demographic, socio-economic and household composition data (age, gender, marital status, employment status, monthly household income, etc.).
- Availability of devices (such as mobile phone, laptop, desktop or fixed PC, tablet, etc.) and household Internet connection.
- Frequency of Internet use, reason for going online, devices used and self-perceived level of Internet proficiency.
- Use of technology for completing processes and administrative operations with social services (degree of perceived difficulty, level of digital autonomy).

### Fieldwork

The fieldwork was carried out between January and July 2022. Face-to-face interviews were conducted in the social services offices of the communities where the research took place.

### Analysis strategy

The initial analyses were descriptive in order to identify sample characteristics and gain a true picture of digitalisation and technology use among the population of social services users. This was based on the different items listed in the description of the instrument.

Bivariate correlations, mean differences and contingency analyses were then carried out. The variables included after the descriptive analysis and the most relevant correlations were harmonised on scales from 1 to 4 for discrete numerical variables and in four categories for categorical nominal variables (barriers and/or limitations, means used and preference of management mode).

The device availability variable was binary coded for the main items available to the population (mobile phone, laptop, desktop PC and tablet) as 0 if none available and 1 if available. This made it possible to calculate an aggregate variable of all devices, the results of which are shown in the following section.

Finally, cluster analysis was used to identify user typologies. Based on the logic proposed by [21], a hierarchical analysis was carried out using the Wards method and the squared Euclidean distance. The criterion used to decide the number of clusters was the visualisation of the dendrogram. The greater explanatory potential of the groups (F) was also determined by the visualisation of the results obtained for the ANOVA analysis. Cluster membership was compared and confirmed by automatic clustering (K-means).

SPSS 27.0 [22] was used to perform the statistical analyses.

## Results

The study findings are reported in accordance with the analytical strategy outlined above. The main descriptive results are presented first, followed by relevant aspects of correlations, contingencies and significant differences. This section concludes by presenting the resulting typology of users and their relationships with key study variables.

### Device availability and household internet connection

This section shows the results for questions on household ownership of various devices and Internet connection.

The most common device is the mobile phone (98.2%), followed by laptops (34.2%) and tablets (28.9%), while the least common are desktop computers (16.7%) and landlines (22.8%).

A total of 40% said they had no Internet connection at home, and 74% said they did not go online outside the home in places with public Internet access (CAPI, libraries, etc.) or through an organisation or association. Of those without Internet access at home, 19.6% go online at some kind of organisation or association.

For the analyses that follow, device ownership was aggregated to give a numerical value from 1 to 4 for household devices (Table 1). This variable aggregates the availability of a mobile phone, laptop, desktop and tablet, with 52% having one device, 19.5% two, 18.6% three and 8% four. Contingency analysis shows that all those with only one device have a mobile phone. Only 1.8% had no device whatsoever (and were excluded from later contingency analyses with this variable).

**Table 1:** Availability of household internet-enabled devices.

			No	DK/DA	Yes	Total
Availability of devices at home	1	Count	37	1	21	59
		% Devices at home	62.7%	1.7%	35.6%	100%
	2	Count	5	0	17	22
		% Devices at home	22.7%	0.0%	77.3%	100%
	3	Count	3	0	18	21
		% Devices at home	14.3%	0.0%	85.7%	100%
	4	Count	0	0	9	9
		% Devices at home	0.0%	0.0%	100%	100%
Total		Count	45	1	65	111
		% Devices at home	40.5%	0.9%	58.6%	100%

As the number of devices increases, the percentage of people who report not having an Internet connection at home falls sharply (contingency coefficient of 0.48; p = .001) (Table 1). Thus, 62.7% of those with only a mobile phone do not have a home Internet connection, while all those with four devices do.

In terms of frequency of Internet use (Table 2), most respondents said they used the Internet every day (62.3%), although 22.8% never or hardly ever do so. The rest use the Internet several times a week (12.3%) or several times a month (2.6%). There is a significant association between the number of devices and frequency of use (contingency coefficient of 0.49; p ≤ .001), with daily Internet use increasing substantially with the number of devices (Table 2). The 42% of people who have only one device say that they never go online. They may have a device, but they do not use it to connect and communicate or carry out processes and administrative operations.

**Table 2:** Device availability with frequency of internet use.

								Total
			Daily	Hardly ever	Never	Several times a week	Several times a month	
Availability of devices / Frequency of Internet use	1	Count	25	4	20	9	1	59
		% Devices	42.4%	6.8%	33.9%	15.3%	1.7%	100%
	2	Count	18	0	0	4	0	22
		% Devices	81.8%	0.0%	0.0%	18.2%	0.0%	100%
	3	Count	19	0	0	1	1	21
		% Devices	90.5%	0.0%	0.0%	4.8%	4.8%	100%
	4	Count	9	0	0	0	0	9
		% Devices	100.0%	0.0%	0.0%	0.0%	0.0%	100%
Total		Count	71	4	20	14	2	111
		% Devices	64.0%	3.6%	18.0%	12.6%	1.8%	100%

### Reason for internet use

In terms of both the reason for and frequency of Internet use, Table 3 shows that most respondents access the Internet very frequently to communicate with family and friends (53.5%), access social networks (37.7%), look for information or news (37.7%) and leisure-related activities (32.5%). Meanwhile, it is rarely used for academic or study purposes, for work-related tasks or for dealing with public or private bodies. Focusing on the use of technology in dealing with public bodies, it is striking how infrequently such resources are used, with only 7% saying they frequently use the Internet for this purpose.

**Table 3:** Frequency and reason for internet use.

Reason	None	A little	Quite often	Very often
Academic work/studies	71.9	0	3.5	6.1
Work	66.7	2.6	2.6	11.4
Manage contacts and jobs	57.9	6.1	6.1	12.3
Communication with family and friends	19.3	4.4	18.4	53.5
Leisure	32.5	16.7	7.9	32.5
Shopping	57	15.8	2.6	2.6
Looking for information/news	34.2	12.3	17.5	37.7
Public bodies	50	17.5	14.9	7
Private entities	51.8	10.5	13.2	15.8
Access to social networks	36	8.8	10.5	37.7

### Use of technology in social services processes and administration

In general, when asked about their self-perception of the type of user they identify with in relation to the Internet and NTs, where 1 is “I don’t know how to use the Internet” and 10 “I consider myself an expert”, the highest percentage of users answered 5 (25.4%), although 22.8% answered 1. The average score is 4.8, indicating a user profile of low-medium proficiency.

As regards user autonomy in carrying out online processes, only 15.8% are able to do so autonomously, 17.5% independently and 17.5% in a combined way, doing some simpler processes on their own and seeking help for others from family and/or friends, government services or information hotlines, associations or community centres in the area. Of the users surveyed, 33% said that they always need help to complete online processes or operations, and the high percentage of users who never carry out any online processes (31.6%) is striking.

In line with these data, only 11.4% of the sample said they had completed a social services process online in the previous year. Of these, 46% found it easy, 30.76% said they had to ask for help but managed to do it correctly, and 23% were unable to do it at all.

To identify the types of processes that users find most difficult, those surveyed were asked about a number of common tasks involved in accessing different benefits and services with social services and how well they were able to deal with them (Table 4). We can see that the process that causes the least difficulty is making an appointment with the service, with 41.2% of users saying that they can manage it easily. However, even in this case, a slightly higher percentage of users feel unable to do so (43%). More than half of users reported feeling unable to perform other tasks, such as renewing benefits, submitting documents electronically, or checking and following up on processes.

**Table 4:** Degree of difficulty in dealing with social services processes (%).

Difficulty of Processes	*Easily*	*I have to ask for help*	*I don’t feel capable*
Service appointment	41.2	10.5	43
Renew social benefit	14.9	21.9	50.9
Submit documentation online	14.9	17.5	55.3
Follow-up on a process	16.7	18.4	52.6

In terms of the methods usually used to carry out some of the most common social services processes (Table 5), face-to-face contact is the most widespread. However, in some cases this is combined with other methods, such as telephone calls, electronic forms or specific applications, e-mail or even instant messaging.

**Table 5:** Preference for the type of service according to the type of process.

	*Face-to-face only*	*Face-to-face and other means*	*Telephone calls*
Request information or appointment	59.6	21.43	12.5
Dependency processes	85.18	7.40	--
Apply for financial aid or economic resources	73.68	15.78	6.57
Electronic purse card	85.71	--	14.28
Large family card	88.88	--	11.11
Access to food programmes	100	--	--
Home help	92.3	--	--
Family/cohabitational intervention	63.63	27.27	9.09
Interviews with professionals	89.42	8.65	--
Submitting documentation	87.61	7.61	0.95
Others (digital certificate/tax declaration)	100	--	--

When asked about their preferred way of dealing with social services, 66.67% of users preferred face-to-face, 26.3% combined, 4.4% by telephone and only 2.6% via the Internet.

### Initial overview of the profile of social services users in relation to the use of NTs

Given the low percentage of users who interact with public bodies using NTs, it would be interesting to determine the characteristics of those who do so. This would help to identify the variables that could influence the varying use of these resources, and thus pinpoint the user profile most likely to use these tools.

Table 6 shows the relationship between a number of key variables and the use of the Internet for dealing with public bodies. Age is a key factor, with a higher percentage of the population carrying out these online tasks in the lower age groups (18–50 years old). In terms of gender, there are no substantial differences in the composition of the groups, with women accounting for 78% of those surveyed. Within each group, of the 79 women who answered this question, 26.6% said they used these resources, compared with 21.05% for men.

**Table 6:** Characteristics of individuals who use the internet to complete processes with public bodies.

Variables	Categories	Yes	No
AGE	18–35	**24**	11.7
	36–50	**32**	24.7
	51–65	44	**46.8**
	65+	--	**16.9**
GENDER	Men	16	24.7
	Women	**84**	75.3
EDUCATION	No education	16	**31.2**
	Primary	36	**55.8**
	Secondary	**28**	11.7
	Higher	**16**	1.3
EMPLOYMENT STATUS	Unemployed	**48**	41.6
	Student	**4**	2.6
	Permanent incapacity	8	10.4
	Household work	12	**23.4**
	Other situation	4	5.2
	Employed work	**20**	5.2
MARITAL STATUS	Marriage	**52**	35.1
	Civil partnership/cohabitation	8	7.8
	Separation/divorced	**24**	19.5
	Single	16	**24.7**
	Widowed	--	**13**
INCOME	Up to 400€	12	**20.8**
	401–800€	48	**57.1**
	1201–1600€	**20**	10.4
	1601–2000€	**8**	--
	2000€+	**12**	3.9

Another of the variables most closely associated with using NTs for these tasks is educational attainment. A higher level of education (secondary and higher) is characteristic of the group using these resources. In terms of employment status, there is a high number of employed people. There are also slightly higher percentages of unemployed people and students. Those working within the household are least likely to use these tools for administrative tasks. In terms of income level, and in line with educational attainment and employment status, people with higher incomes are more likely to belong to the first group that completes processes online.

Correlation analyses were performed (Table 7) to explore in more detail the variables that seem to influence the varying degrees of Internet use when dealing with public bodies. Age, educational attainment and income were found to be significant variables. In other words, higher levels of education increase the likelihood of using these resources. The correlation is also positive and significant for monthly household income. Age has a negative correlation, i.e. the older the person, the lower the likelihood of using the Internet for dealing with public bodies.

**Table 7:** Correlations: Carrying out processes with public bodies.

		Carrying out Processes with Public Bodies (Internet)	Age	Educational Attainment	Type of Internet and NTs user	Monthly household income
Carrying out Processes with Public Bodies (Internet)	Pearson Correlation	1	**-.250** ^*^	**.470** ^**^	**.485** ^**^	**.307** ^**^
	Sig. (bilateral)		.011	.000	.000	.002
	N	102	102	102	102	96

*. The correlation is significant at the 0.05 level (bilateral).

**. The correlation is significant at the 0.01 level (bilateral).

The analysis includes another variable called “Type of Internet and NTs user”. Respondents were invited to rate themselves on a scale from 1 to 10 (the higher the score, the more they use these tools). This variable is also significantly and positively correlated. In other words, the greater the mastery of these tools, the more technology is used to carry out processes with public bodies.

If we look at the profile of users who use the Internet to deal with public bodies, they tend to be younger, more likely to have completed secondary or higher education and to have a higher income. Users who perform these operations online are also identified as having a higher level of mastery of NTs.

So far, we have analysed the variables that influence the varying degrees of technology use when dealing with public bodies. The next step is to analyse the impact of these variables when it comes to handling specific social services processes online, such as making appointments, social benefits, submitting documentation electronically, and following up on processes (Table 8). The original response categories included the following options: performed the operation with ease, needed help or felt unable to perform the operation. This variable has been recoded by dropping the option where help is needed in order to obtain two more distinct categories: those who can perform the operation with ease and those who do not feel able to do so.

**Table 8:** Correlations: Social services processes.

		Age	Educational Attainment	Type of Internet and NTs user	Processes with Public Bodies (Internet)	Monthly household income
Making service appointment	Pearson Correlation	**-.412** ^**^	**.302** ^**^	**.654** ^**^	**.656** ^**^	**.243** ^*^
	Sig. (bilateral)	.000	.003	.000	.000	.020
	N	96	96	96	86	91
Apply to renew social benefits	Pearson Correlation	**-.350** ^**^	**.425** ^**^	**.573** ^**^	**.660** ^**^	**.354** ^**^
	Sig. (bilateral)	.002	.000	.000	.000	.002
	N	75	75	75	67	71
Submit documentation online	Pearson Correlation	**-.337** ^**^	**.462** ^**^	**.529** ^**^	**.581** ^**^	**.317** ^**^
	Sig. (bilateral)	.002	.000	.000	.000	.005
	N	80	80	80	71	76
Following-up the on process	Pearson Correlation	**-.312** ^**^	**.416** ^**^	**.529** ^**^	**.704** ^**^	**.351** ^**^
	Sig. (bilateral)	.005	.000	.000	.000	.002
	N	79	79	79	70	75

**. The correlation is significant at the 0.01 level (bilateral).

*. The correlation is significant at the 0.05 level (bilateral).

Correlation analysis was then carried out with age, educational attainment, monthly income and self-perceived ability to use NTs in dealing with public bodies.

These variables again correlate significantly in the case of specific social services processes. For age, the correlation coefficients are quite similar, although slightly higher for making an appointment. Educational attainment shows a slightly lower correlation coefficient for making an appointment, which seems to indicate a lower level of difficulty or less need for training in using this tool. The highest coefficient is found for submitting documentation online. The better people feel about using technology, the more likely they are to be able to carry out these processes with ease. Similarly, the more familiar people are with completing processes with public bodies online, the more likely they are to do so easily. In this case, the highest correlation coefficient is found for checking and following up on processes. For the “monthly household income” variable, very similar coefficients are observed for all processes, except the first one involving making an appointment.

### User typology based on mastery of NTs and relationship with social services

We now have an initial overview of the variables that seem to explain the varying levels of mastery of NTs, and hence how they are used to perform various administrative operations with public bodies, and the perceived degree of difficulty in using them. As such, it would now be interesting to find out the types of users involved.

Given the descriptive results, associations and correlations found, the following variables were included in this user classification analysis (clusters), using hierarchical classification and the Wards method (Table 9): age, educational attainment, self-perceived level of using NTs, the degree of autonomy shown in carrying out these processes, barriers to carrying out these processes and the varying availability of devices for going online at home. Given the higher number of missing cases for income level and the use of the Internet for dealing with public bodies, these variables have been excluded. All variables included in the model have been standardised on a scale from 1–4.

**Table 9:** ANOVA of variables included in the user typology.

		Sum of squares	gl	Root mean square	F	Sig.
Age	Inter-groups	28.567	2	14.283	29.786	.000
	Intra-groups	50.350	105	.480		
	Total	78.917	107			
Educational attainment	Inter- groups	6.726	2	3.363	5.422	.006
	Intra- groups	65.125	105	.620		
	Total	71.852	107			
Internet user type NT	Inter- groups	54.409	2	27.204	43.063	.000
	Intra- groups	66.332	105	.632		
	Total	120.741	107			
Barriers to Internet processes	Inter- groups	83.322	2	41.661	237.501	.000
	Intra- groups	18.419	105	.175		
	Total	101.741	107			
Availability of devices	Inter- groups	34.257	2	17.128	21.922	.000
	Intra- groups	82.039	105	.781		
	Total	116.296	107			
Degree of autonomy for online processes	Inter- groups	63.878	2	31.939	58.181	.000
	Intra- groups	57.641	105	.549		
	Total	121.519	107			

The result of this process is a user classification based on the degree of similarity between them, taking into account each of the variables included. In the end, three clusters were obtained. The one-factor ANOVA analysis suggests statistically significant differences for all the variables included.

The characterisation of the extracted clusters reveals three very clearly differentiated types of social services users (Table 10), mainly among the users included in Clusters 2 and 3.

**Table 10:** Types of social services users.

		Cluster 1 (37)	Cluster 2 (35)	Cluster 3 (36)
Age	18–35 years old	24.3	--	19.4
	36 50 years old	51.4	2.9	41.7
	51–65 years old	24.3	**74.3**	33.3
	65+	--	**22.9**	5.6
	***Age Average***	***42*.*51***	***60*.*97***	***46*.*31***
Educational Attainment	No education	32.4	25.7	16.7
	Primary	54.1	60	22.2
	Secondary	10.8	14.3	**22.2**
	University and Higher education	2.7	--	**16.7**
Internet user type (self-perception)	Low	13.5	**74.3**	2.8
	Medium-low	43.2	22.9	27.8
	Medium-high	27	2.9	**36.1**
	High	16.2	--	**33.3**
	***Average***	***5*.*68***	***2*.*23***	***6*.*86***
Handling processes (Internet)	Does not carry out any processes	40.5	**54.3**	--
	Needs help	43.2	40	16.7
	Combined: autonomy and assistance	10.8	5.7	**38.9**
	Autonomy	5.4	--	**44.4**
Barriers to the management of Internet processes	No devices available	2.7	**8.6**	--
	Lack of knowledge of use	78.4	74.3	--
	Lack of interest	16.2	17.1	--
	Does not identify barrier	2.7	--	100
Availability of devices	1 device	54.1	**85.7**	19.4
	2 devices	13.5	14.3	30.6
	3 devices	24.3	--	**33.3**
	4 devices	8.1	--	**16.7**
Household income	Up to 400€	24.2	**25.7**	14.7
	401–800€	57.6	**62.9**	50
	1200–1600€	12.1	11.4	**17.6**
	1600€+	6.1	--	**17.6**
*Processes with public bodies*	None	66.7	**93.9**	2.9
	A little	10	6.1	44.1
	Quite often	23.3	--	**29.4**
	Very often	--	--	**23.5**

Cluster 3 is characterised by the fact that it includes those users who are better placed in terms of several variables and their use of technology and online processes. These are users with an average age of 46, greater educational attainment (secondary and higher), higher incomes and who identify themselves as higher users of the Internet and NTs (6.86). People in this typology therefore have a higher degree of autonomy in dealing with social service processes online, with no barriers to doing so. These are people with more devices for using the Internet at home and who carry out online processes with public bodies more often.

Cluster 2 is characterised by the fact that it includes older users (60.97 years old), with a high percentage of people with no education or only primary education, lower income levels and who are mainly identified as people with low Internet and NT usage, with an average for this indicator of 2.23 for this group. In this respect, more than half of the people falling into this typology do not perform online processes and, if they do, they need help. In terms of barriers to performing such tasks, 8.6% said that they did not have suitable devices and just over 74% did not know how to use them. A ***total*** of 85.7% have only one device for connecting to the Internet at home. This is always a mobile phone, which cannot be used to perform tasks that require, for example, a digital certificate. Consequently, almost 94% of this group do not use the Internet to deal with public bodies.

In the middle ground are those users falling into Cluster 1. They are identified as those with the lowest average age (42.5 years) and the highest percentage of people with no education. Unlike Cluster 2, however, this group contains a small percentage of people with a higher level of education. Around 82% earn at most 800€, although 18% earn from 1200–1600€ or above.

People in this typology are in the middle range (5.68) in terms of where they place themselves when it comes to their level of Internet and NT usage. Nevertheless, 40% do not carry out online processes and, if they do, they need help (43.2%). The main barriers to completing social services processes online were lack of knowledge and disinterest. With regard to the availability of devices in the home, the highest percentage reported having only one device (54%), although there are households with two, three or four. Also noteworthy is the high percentage of people who do not use the Internet to deal with public bodies (66.7%), although 23% use it extensively.

## Discussion

The findings have revealed a situation in which social services users have high levels of unemployment and a high number of people who are inactive in the labour market. With 7 out of 10 people living on less than 800€ per month, the economic reality is one of high vulnerability. Most are women, with an average age of 50. It is likely that these characteristics directly relate to the general profile of people who use social services most, where it is traditionally women who apply for social support and social intervention services. This is perhaps even more so in countries associated with traditional Mediterranean and familist welfare state models, where the care and management of domestic issues has fallen to women. This also ties in with the economic and labour market realities described above.

This analysis of associations and correlations, along with the profile of social services users in terms of how they use technologies and interact with services, shows that there is still work to be done and considerable room for improvement.

This reality has previously been articulated and poses challenges that go beyond digital divides. It also guarantees personal and professional digital rights, in a context of mainstreaming technology within relationships, service delivery and the transformation of professional interventions.

Generally speaking, social services users are not familiar with using NTs to carry out administrative tasks and prefer to do so in person. Without doubt, this has to do with the population profile, characterised by an older age group, low-medium educational attainment and low-medium usage of these tools. However, as we have seen, there are different types of users. In designing strategies to promote the use of NTs in dealing with social services, these must be tailored to the user profile. Similarly, interventions using these tools will undoubtedly need a great deal of work with the population. They will also require liaison with professionals and the system itself.

It is likely that there will be a proportion of the population with whom it will be more difficult to achieve the targets set for the digitalisation of social services (Cluster 2). This is because changing existing habits and overcoming skills gaps in using these tools will be more difficult. Such users are older and, despite having a mobile phone, do not have or use it for managing their lives, but rather as a means of family or social communication. Furthermore, it is not always feasible to successfully perform the system’s operations and processes with a mobile phone type device.

However, by adapting the tools to people’s needs, and tailoring the training offered in this regard, significant advances can be made in terms of levels of use and handling of NTs for dealing with social services in much of the user population, here mostly identified as Types 1 and 3. Such improvements must bear in mind both the type of user and the availability of devices. Most of the population has a mobile phone, but using one to carry out administrative operations and transactions is not always feasible.

## Conclusions

In recent years, there has been a growing trend towards using technology in connection with social services, along with users’ willingness to use it in their dealings with the system. People feel more comfortable using technology for a variety of tasks, such as accessing information, filling in forms and communicating with public bodies. However, a significant proportion of the population, particularly older people and people in rural areas, may not have access to or be comfortable with technology [23]. This can make it difficult for these people to access information and services, and highlights the need for social services organisations to provide a range of options for accessing services, including face-to-face and over the phone.

The willingness and readiness to use technology in social services processes is therefore on the rise, but significant challenges remain in ensuring that all users have access to and feel comfortable using these tools. Social services agencies must continue to invest in digital tools and provide a variety of options for accessing services that meet the needs of all users.

Finally, much work remains on the use of NTs when dealing with public bodies and, more specifically, with social services. As part of this work, the framework project of this research is working on an appraisal of the social services systems, professionals and users at a local level, in order to complete this profile. This project is also working to identify and guide actions that, by integrating technology, will bring about digital innovation in services and improved access for the population. A co-diagnosis and co-design Hubs approach is being taken to develop processes that enable the digitalisation of social services through innovation. This involves adapting measures and change processes to ensure high-quality service delivery in the digital era, in collaboration with professionals, users and the public systems and services themselves.
